# 4-[(2-Hy­droxy­benzyl­idene)amino]-*N*-(5-methyl­isoxazol-3-yl)benzene­sulfonamide: a monoclinic polymorph

**DOI:** 10.1107/S1600536810036585

**Published:** 2010-09-18

**Authors:** Samuel Ebenezer, Packianathan Thomas Muthiah

**Affiliations:** a School of Chemistry, Bharathidasan University, Tiruchirappalli 620 024, Tamilnadu, India

## Abstract

The title compound, C_17_H_15_N_3_O_4_S, is a monoclinic polymorph with space group *P*2_1_/*c* of the previously reported triclinic form in *P* 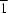 [Subashini *et al.* (2009 ▶). *J. Chem. Crystallogr.* **39**, 112–116]. In both polymorphs, intra­molecular O—H⋯N hydrogen bonds and dimer formation *via* a pair of inter­molecular N—H⋯N hydrogen bonds with an *R*_2_^2^(8) motif are observed. The two polymorphs differ in the next level of supra­molecular organization involving C—H⋯O hydrogen bonds with varied packing and different conformations.

## Related literature

For the biological relevance of sulfonamide drugs and their Schiff base derivatives, see: Genc *et al.* (2008 ▶); Supuran *et al.* (1997 ▶). For the triclinic polymorph of the title compound, see: Subashini *et al.* (2009 ▶). For 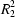
(8) ring motifs in sulfonamides, see: Adsmond & Grant (2001 ▶). For conformational studies on sulfonamides, see: Kálmán *et al.* (1981 ▶).
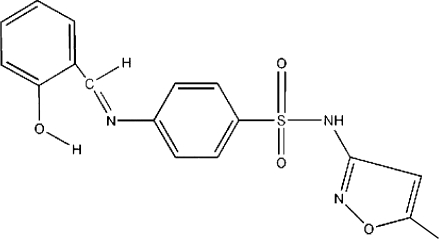


## Experimental

### 

#### Crystal data

C_17_H_15_N_3_O_4_S
*M* *_r_* = 357.39
Monoclinic, 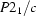

*a* = 7.0374 (1) Å
*b* = 17.9244 (3) Å
*c* = 14.5175 (3) Å
β = 112.962 (1)°
*V* = 1686.15 (5) Å^3^
*Z* = 4
Mo *K*α radiation
μ = 0.22 mm^−1^
*T* = 293 K
0.25 × 0.22 × 0.20 mm

#### Data collection

Bruker SMART APEXII CCD area-detector diffractometer
Absorption correction: multi-scan (*SADABS*; Bruker, 2008 ▶) *T*_min_ = 0.947, *T*_max_ = 0.957
14935 measured reflections
2953 independent reflections
2198 reflections with *I* > 2σ(*I*)
*R*_int_ = 0.038

#### Refinement

*R*[*F* ^2^ > 2σ(*F* ^2^)] = 0.049
*wR*(*F* ^2^) = 0.115
*S* = 1.06
2953 reflections
227 parameters
H-atom parameters constrained
Δρ_max_ = 0.25 e Å^−3^
Δρ_min_ = −0.30 e Å^−3^

### 

Data collection: *APEX2* (Bruker, 2008 ▶); cell refinement: *SAINT* (Bruker, 2008 ▶); data reduction: *SAINT*; program(s) used to solve structure: *SHELXS86* (Sheldrick, 2008 ▶); program(s) used to refine structure: *SHELXL97* (Sheldrick, 2008 ▶); molecular graphics: *PLATON* (Spek, 2009 ▶); software used to prepare material for publication: *PLATON*.

## Supplementary Material

Crystal structure: contains datablocks global, I. DOI: 10.1107/S1600536810036585/is2595sup1.cif

Structure factors: contains datablocks I. DOI: 10.1107/S1600536810036585/is2595Isup2.hkl

Additional supplementary materials:  crystallographic information; 3D view; checkCIF report

## Figures and Tables

**Table 1:** Hydrogen-bond geometry (Å, °)

*D*—H⋯*A*	*D*—H	H⋯*A*	*D*⋯*A*	*D*—H⋯*A*
O1—H1⋯N1	0.82	1.88	2.606 (3)	147
N2—H2*A*⋯N3^i^	0.86	2.24	2.898 (4)	134
C10—H10⋯O1^ii^	0.93	2.53	3.313 (3)	141

Symmetry codes: (i) 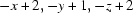; (ii) 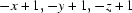.

**Fig. 1.**
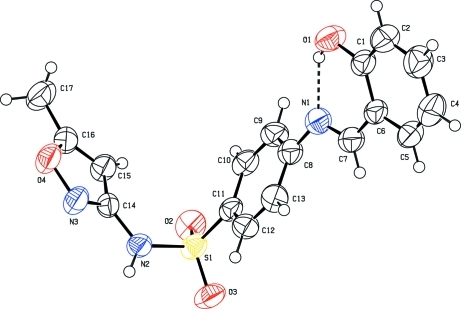
An ORTEP view of new dimorph showing 50% probability displacement ellipsoids.

**Fig. 2.**
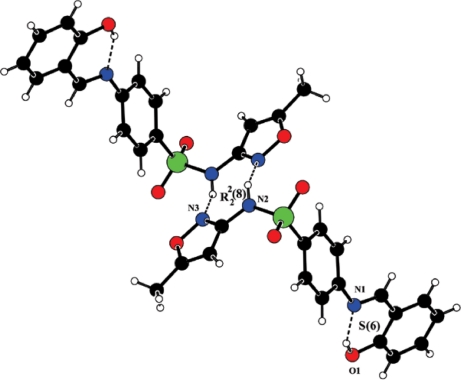
Two inversely related molecules form dimer through R22(8) motif. [Symmetry code: (i) -x + 2, -y + 1, -z + 2.]

**Fig. 3.**
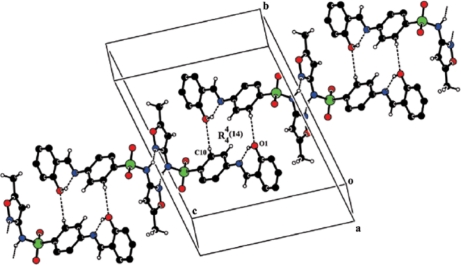
View of supramolecular chain formed through the dimers via weak C—H···O interactions.

## supplementary crystallographic information

### Comment

Sulfonamide drugs are known to have antibacterial, antifungal,antimalarial and antileprotic properties (Genc *et al.*, 2008). Schiff bases derived from sulfonamide compounds also display enzymatic inhibition (Supuran *et al.*, 1997). A polymorph, I, of the title compound has been reported earlier from our group (Subashini *et al.*, 2009). In the present study we report a new dimorph, II, and its crystal structure.

The new dimorph crystallizes in monoclinic system (*P*2_1_/*c*) which contains only one molecule in the asymmetric unit (Fig. 1). There is one N—H···N intermolecular hydrogen bond [N2···N3 = 2.899 (3) Å; Table 1], where the amido nitrogen acts as a donor and the nitrogen of the isoxazole acts as an acceptor. The donor and acceptor of one of the Schiff base molecule pair up with another inversely related molecule to form a dimer with graph set notation *R*_2_^2^(8) (Fig 2). A similar type of homosynthon is also formed in the polymorph I. A recent statistical survey has shown that the absence of amino group in the sulfonamide compounds further encourages amido protons to hydrogen bond with an activated nitrogen (atom of a heterocycle that lies in conjugation with the amido nitrogen) of the heterocyclic ring to form an optimum sized *R*_2_^2^(8) ring motif (Adsmond & Grant, 2001). This could be the reason why both the polymorphs form the same synthons thereby succumb to similar first order arrangement of molecules.

Polymorph II forms an intramolecular C—H···O bond with a graph set notation S(6) similar to its predecessor. It is only the weaker bonds in both the polymorphs that are significantly responsible for the differences in arrangement of molecules in the crystal. In both the polymorphs the homosynthon combines with each other through a couple of weak C—H···O bonds (C10—H10···O1 in polymorph II) forming a large ring motif with graph set notation *R*_4_^4^(12) and *R*_4_^4^(14) for polymorphs I and II respectively. These homosynthons stretch along a particular direction linked through weak C—H···O interaction forming a supramolecular chain (Fig. 3).

In addition to the packing differences there are changes in the conformations of the molecules which are obvious from their torsion angles. Though there are many possible conformations in which the molecule could exist, the steric and electrostatic constraints of sulfonamides have restricted them to torsion angles between 70–120° and 60–90° for ε1 and ε2 respectively [where ε1 and ε2 are the modulus of the torsion angles along C—S and S—N bonds respectively] (Kálmán *et al.*, 1981). The angles ε1 and ε2 for polymorph II lie in the expected range. The bond lengths and the bond angles among the polymorphs are more or less close to one another but differ hugely in their torsion angles.

To conclude with, both dimorphs posses same intermolecular hydrogen bonds leading to formation of dimers through N—H···N hydrogen bonds, showing similar first level of organization. They differ only in the higher level supramolecular organization through C—H···O hydrogen bonds. A thorough inspection reveals that conformational changes in the molecule lead to the packing differences between the dimorphs.

### Experimental

The base of the title compound was synthesized by refluxing ethanolic solutions of 4-amino-*N*-(5-methyl-3-isoxazolyl) benzene sulfonamide (SMZ) (25 mg, Qualigens) with 2-hydroxy benzaldehyde (SA) (25 mg, LOBA Chemie) in an equimolar ratio. The mixture was refluxed for up to 6 h and then transferred to a beaker which was eventually allowed to evaporate slowly in the mother liquor. After a couple of days yellow crystals separated out. This base was characterized using single-crystal XRD method and found to be a new polymorph.

### Refinement

All hydrogen atoms were positioned geometrically and were refined using riding model. The C—H, N—H and O—H bond lengths are 0.93–0.97, 0.86 and 0.82 Å, respectively. [*U*_iso_(H) = 1.2*U*_eq_(aromatic C and N) and 1.5*U*_eq_(methyl C and O)].

### Crystal data

**Table:**

C_17_H_15_N_3_O_4_S	*F*(000) = 744
*M**_r_* = 357.39	*D*_x_ = 1.408 Mg m^−^^3^
Monoclinic, *P*2_1_/*c*	Mo *K*α radiation, λ = 0.71073 Å
Hall symbol: -P 2ybc	Cell parameters from 2953 reflections
*a* = 7.0374 (1) Å	θ = 1.9–25.0°
*b* = 17.9244 (3) Å	µ = 0.22 mm^−^^1^
*c* = 14.5175 (3) Å	*T* = 293 K
β = 112.962 (1)°	PRISM, yellow
*V* = 1686.15 (5) Å^3^	0.25 × 0.22 × 0.20 mm
*Z* = 4	

### Data collection

**Table:**

Bruker SMART APEXII CCD area-detector diffractometer	2953 independent reflections
Radiation source: fine-focus sealed tube	2198 reflections with *I* > 2σ(*I*)
graphite	*R*_int_ = 0.038
φ and ω scans	θ_max_ = 25.0°, θ_min_ = 1.9°
Absorption correction: multi-scan (*SADABS*; Bruker, 2008)	*h* = −7→8
*T*_min_ = 0.947, *T*_max_ = 0.957	*k* = −19→21
14935 measured reflections	*l* = −17→17

### Refinement

**Table:**

Refinement on *F*^2^	Primary atom site location: structure-invariant direct methods
Least-squares matrix: full	Secondary atom site location: difference Fourier map
*R*[*F*^2^ > 2σ(*F*^2^)] = 0.049	Hydrogen site location: inferred from neighbouring sites
*wR*(*F*^2^) = 0.115	H-atom parameters constrained
*S* = 1.06	*w* = 1/[σ^2^(*F*_o_^2^) + (0.040*P*)^2^ + 0.9817*P*] where *P* = (*F*_o_^2^ + 2*F*_c_^2^)/3
2953 reflections	(Δ/σ)_max_ < 0.001
227 parameters	Δρ_max_ = 0.25 e Å^−^^3^
0 restraints	Δρ_min_ = −0.30 e Å^−^^3^

### Special details

**Table:**

Geometry. Bond distances, angles *etc*. have been calculated using the rounded fractional coordinates. All su's are estimated from the variances of the (full) variance-covariance matrix. The cell e.s.d.'s are taken into account in the estimation of distances, angles and torsion angles
Refinement. Refinement on *F*^2^ for ALL reflections except those flagged by the user for potential systematic errors. Weighted *R*-factors *wR* and all goodnesses of fit *S* are based on *F*^2^, conventional *R*-factors *R* are based on *F*, with *F* set to zero for negative *F*^2^. The observed criterion of *F*^2^ > σ(*F*^2^) is used only for calculating -*R*-factor-obs *etc*. and is not relevant to the choice of reflections for refinement. *R*-factors based on *F*^2^ are statistically about twice as large as those based on *F*, and *R*-factors based on ALL data will be even larger.

### Fractional atomic coordinates and isotropic or equivalent isotropic displacement parameters (Å^2^)

**Table:**

	*x*	*y*	*z*	*U*_iso_*/*U*_eq_	
S1	0.61583 (11)	0.37355 (3)	0.87561 (5)	0.0507 (3)	
O1	0.8540 (3)	0.47399 (10)	0.37502 (16)	0.0676 (8)	
O2	0.3998 (3)	0.38398 (10)	0.84707 (15)	0.0625 (7)	
O3	0.7159 (3)	0.30911 (10)	0.93038 (15)	0.0667 (8)	
O4	0.7150 (3)	0.63662 (10)	0.93497 (15)	0.0658 (7)	
N1	0.8210 (4)	0.39344 (12)	0.51746 (17)	0.0551 (8)	
N2	0.7352 (3)	0.44313 (11)	0.94686 (16)	0.0501 (8)	
N3	0.8188 (4)	0.56808 (12)	0.96244 (18)	0.0594 (9)	
C1	0.9847 (4)	0.42129 (14)	0.3684 (2)	0.0528 (10)	
C2	1.0800 (5)	0.43213 (17)	0.3024 (2)	0.0664 (11)	
C3	1.2136 (6)	0.38013 (19)	0.2939 (3)	0.0770 (14)	
C4	1.2539 (6)	0.31558 (19)	0.3500 (3)	0.0787 (16)	
C5	1.1614 (5)	0.30449 (15)	0.4157 (2)	0.0650 (11)	
C6	1.0262 (4)	0.35658 (13)	0.4270 (2)	0.0495 (9)	
C7	0.9426 (4)	0.34594 (15)	0.5023 (2)	0.0549 (10)	
C8	0.7705 (4)	0.38551 (14)	0.6022 (2)	0.0506 (9)	
C9	0.5773 (4)	0.40732 (14)	0.5957 (2)	0.0544 (10)	
C10	0.5264 (4)	0.40347 (14)	0.6783 (2)	0.0511 (10)	
C11	0.6728 (4)	0.37909 (13)	0.7690 (2)	0.0459 (8)	
C12	0.8681 (4)	0.35926 (15)	0.7762 (2)	0.0544 (10)	
C13	0.9170 (4)	0.36252 (15)	0.6943 (2)	0.0561 (10)	
C14	0.6769 (4)	0.51741 (13)	0.92693 (19)	0.0447 (9)	
C15	0.4810 (4)	0.54900 (15)	0.8754 (2)	0.0543 (10)	
C16	0.5134 (4)	0.62267 (15)	0.8831 (2)	0.0551 (10)	
C17	0.3782 (5)	0.68894 (17)	0.8475 (3)	0.0798 (13)	
H1	0.81330	0.46250	0.41890	0.1010*	
H2	1.05290	0.47510	0.26360	0.0800*	
H2A	0.84130	0.43320	1.00030	0.0600*	
H3	1.27820	0.38830	0.24980	0.0920*	
H4	1.34340	0.27990	0.34300	0.0940*	
H5	1.18960	0.26110	0.45380	0.0780*	
H7	0.97800	0.30310	0.54140	0.0660*	
H9	0.48080	0.42480	0.53520	0.0650*	
H10	0.39510	0.41710	0.67330	0.0610*	
H12	0.96640	0.34360	0.83730	0.0650*	
H13	1.04890	0.34940	0.69990	0.0670*	
H15	0.35650	0.52420	0.84310	0.0650*	
H17A	0.39730	0.71040	0.79110	0.1200*	
H17B	0.41320	0.72510	0.90030	0.1200*	
H17C	0.23670	0.67420	0.82810	0.1200*	

### Atomic displacement parameters (Å^2^)

**Table:**

	*U*^11^	*U*^22^	*U*^33^	*U*^12^	*U*^13^	*U*^23^
S1	0.0458 (4)	0.0386 (4)	0.0611 (5)	−0.0045 (3)	0.0136 (3)	0.0006 (3)
O1	0.0703 (14)	0.0556 (12)	0.0757 (14)	0.0180 (10)	0.0273 (12)	0.0130 (10)
O2	0.0397 (11)	0.0645 (12)	0.0791 (14)	−0.0079 (9)	0.0186 (10)	−0.0030 (10)
O3	0.0735 (14)	0.0420 (10)	0.0787 (15)	0.0032 (10)	0.0233 (12)	0.0149 (10)
O4	0.0604 (13)	0.0418 (10)	0.0777 (14)	0.0020 (9)	0.0081 (11)	−0.0114 (10)
N1	0.0494 (14)	0.0503 (13)	0.0569 (15)	−0.0036 (11)	0.0112 (12)	−0.0023 (11)
N2	0.0453 (13)	0.0437 (12)	0.0482 (14)	0.0015 (10)	0.0040 (10)	0.0004 (10)
N3	0.0514 (15)	0.0416 (13)	0.0701 (17)	0.0036 (11)	0.0072 (12)	−0.0079 (11)
C1	0.0501 (17)	0.0427 (15)	0.0569 (18)	−0.0005 (13)	0.0115 (14)	−0.0037 (13)
C2	0.075 (2)	0.0521 (18)	0.072 (2)	−0.0019 (16)	0.0286 (18)	0.0057 (15)
C3	0.093 (3)	0.070 (2)	0.081 (2)	0.0014 (19)	0.048 (2)	−0.0028 (18)
C4	0.086 (3)	0.063 (2)	0.092 (3)	0.0162 (18)	0.040 (2)	−0.0062 (19)
C5	0.074 (2)	0.0438 (16)	0.066 (2)	0.0054 (15)	0.0150 (17)	−0.0016 (14)
C6	0.0493 (16)	0.0375 (14)	0.0508 (17)	−0.0035 (12)	0.0077 (13)	−0.0032 (12)
C7	0.0539 (18)	0.0430 (15)	0.0542 (18)	−0.0101 (13)	0.0062 (14)	−0.0002 (13)
C8	0.0494 (17)	0.0421 (14)	0.0530 (18)	−0.0076 (12)	0.0120 (14)	−0.0018 (13)
C9	0.0432 (17)	0.0520 (16)	0.0547 (18)	0.0016 (13)	0.0048 (14)	0.0039 (13)
C10	0.0371 (15)	0.0427 (15)	0.066 (2)	0.0016 (12)	0.0121 (14)	0.0005 (13)
C11	0.0417 (15)	0.0334 (13)	0.0529 (16)	−0.0021 (11)	0.0080 (13)	−0.0019 (12)
C12	0.0446 (17)	0.0538 (16)	0.0513 (18)	0.0040 (13)	0.0042 (14)	0.0002 (13)
C13	0.0401 (16)	0.0588 (18)	0.061 (2)	0.0018 (13)	0.0105 (14)	−0.0047 (14)
C14	0.0452 (16)	0.0420 (14)	0.0426 (16)	0.0005 (12)	0.0125 (13)	−0.0054 (12)
C15	0.0433 (16)	0.0483 (16)	0.0607 (19)	0.0027 (12)	0.0087 (14)	−0.0045 (13)
C16	0.0545 (18)	0.0513 (17)	0.0512 (17)	0.0068 (14)	0.0115 (14)	−0.0074 (13)
C17	0.078 (2)	0.0523 (18)	0.090 (3)	0.0170 (16)	0.012 (2)	−0.0013 (17)

### Geometric parameters (Å, °)

**Table:**

S1—O2	1.424 (2)	C8—C9	1.382 (4)
S1—O3	1.423 (2)	C9—C10	1.381 (4)
S1—N2	1.629 (2)	C10—C11	1.388 (4)
S1—C11	1.747 (3)	C11—C12	1.384 (4)
O1—C1	1.348 (3)	C12—C13	1.362 (4)
O4—N3	1.405 (3)	C14—C15	1.406 (4)
O4—C16	1.345 (4)	C15—C16	1.337 (4)
O1—H1	0.8200	C16—C17	1.483 (4)
N1—C7	1.286 (4)	C2—H2	0.9300
N1—C8	1.415 (4)	C3—H3	0.9300
N2—C14	1.390 (3)	C4—H4	0.9300
N3—C14	1.299 (4)	C5—H5	0.9300
N2—H2A	0.8600	C7—H7	0.9300
C1—C2	1.381 (4)	C9—H9	0.9300
C1—C6	1.400 (4)	C10—H10	0.9300
C2—C3	1.363 (5)	C12—H12	0.9300
C3—C4	1.379 (5)	C13—H13	0.9300
C4—C5	1.363 (5)	C15—H15	0.9300
C5—C6	1.388 (4)	C17—H17A	0.9600
C6—C7	1.442 (4)	C17—H17B	0.9600
C8—C13	1.395 (4)	C17—H17C	0.9600
			
S1···H15	3.1900	C2···H15^ii^	3.0700
O1···N1	2.606 (3)	C5···H12^ix^	3.0000
O1···N1^i^	3.245 (3)	C7···H13	2.6700
O1···C10^ii^	3.313 (3)	C7···H1	2.4100
O2···C13^iii^	3.276 (4)	C13···H7	2.6400
O2···C15	3.010 (3)	C13···H2^i^	2.9600
O3···C7^iv^	3.172 (3)	C16···H3^i^	2.8500
O1···H10^ii^	2.5300	H1···N1	1.8800
O2···H10	2.5800	H1···C7	2.4100
O2···H15	2.5300	H2···C13^i^	2.9600
O2···H13^iii^	2.6300	H2A···N3^vii^	2.2400
O3···H12	2.6800	H3···O4^i^	2.7400
O3···H4^v^	2.9000	H3···C16^i^	2.8500
O3···H7^iv^	2.7800	H4···O3^x^	2.9000
O4···H5^vi^	2.6800	H5···H7	2.4200
O4···H3^i^	2.7400	H5···O4^xi^	2.6800
N1···O1	2.606 (3)	H7···C13	2.6400
N1···O1^i^	3.245 (3)	H7···H5	2.4200
N2···N3^vii^	2.898 (4)	H7···H13	2.3100
N3···N2^vii^	2.898 (4)	H7···O3^ix^	2.7800
N1···H1	1.8800	H10···O2	2.5800
N3···H2A^vii^	2.2400	H10···O1^ii^	2.5300
C2···C8^i^	3.546 (4)	H12···O3	2.6800
C5···C9^viii^	3.581 (4)	H12···C5^iv^	3.0000
C7···O3^ix^	3.172 (3)	H13···O2^viii^	2.6300
C8···C2^i^	3.546 (4)	H13···C7	2.6700
C9···C5^iii^	3.581 (4)	H13···H7	2.3100
C10···O1^ii^	3.313 (3)	H15···S1	3.1900
C13···O2^viii^	3.276 (4)	H15···O2	2.5300
C15···O2	3.010 (3)	H15···C2^ii^	3.0700
C2···H17C^ii^	2.9800	H17C···C2^ii^	2.9800
			
O2—S1—O3	120.58 (13)	N3—C14—C15	111.9 (2)
O2—S1—N2	108.18 (12)	N2—C14—N3	117.9 (3)
O2—S1—C11	108.84 (13)	N2—C14—C15	130.2 (2)
O3—S1—N2	104.27 (12)	C14—C15—C16	104.7 (2)
O3—S1—C11	107.86 (13)	O4—C16—C15	109.8 (2)
N2—S1—C11	106.23 (12)	O4—C16—C17	116.1 (2)
N3—O4—C16	108.3 (2)	C15—C16—C17	134.1 (3)
C1—O1—H1	110.00	C1—C2—H2	120.00
C7—N1—C8	119.5 (2)	C3—C2—H2	120.00
S1—N2—C14	124.33 (18)	C2—C3—H3	120.00
O4—N3—C14	105.4 (2)	C4—C3—H3	120.00
S1—N2—H2A	118.00	C3—C4—H4	120.00
C14—N2—H2A	118.00	C5—C4—H4	120.00
C2—C1—C6	119.7 (3)	C4—C5—H5	119.00
O1—C1—C6	121.6 (3)	C6—C5—H5	119.00
O1—C1—C2	118.7 (2)	N1—C7—H7	119.00
C1—C2—C3	120.4 (3)	C6—C7—H7	119.00
C2—C3—C4	120.7 (4)	C8—C9—H9	120.00
C3—C4—C5	119.4 (4)	C10—C9—H9	120.00
C4—C5—C6	121.4 (3)	C9—C10—H10	120.00
C5—C6—C7	120.1 (2)	C11—C10—H10	120.00
C1—C6—C5	118.4 (3)	C11—C12—H12	120.00
C1—C6—C7	121.3 (2)	C13—C12—H12	120.00
N1—C7—C6	122.5 (2)	C8—C13—H13	120.00
N1—C8—C9	119.1 (2)	C12—C13—H13	120.00
N1—C8—C13	121.4 (3)	C14—C15—H15	128.00
C9—C8—C13	119.3 (3)	C16—C15—H15	128.00
C8—C9—C10	120.5 (3)	C16—C17—H17A	109.00
C9—C10—C11	119.4 (3)	C16—C17—H17B	109.00
S1—C11—C12	118.9 (2)	C16—C17—H17C	109.00
S1—C11—C10	121.1 (2)	H17A—C17—H17B	109.00
C10—C11—C12	120.0 (3)	H17A—C17—H17C	110.00
C11—C12—C13	120.3 (3)	H17B—C17—H17C	109.00
C8—C13—C12	120.4 (3)		
			
O2—S1—N2—C14	−44.7 (3)	C2—C1—C6—C5	−0.8 (4)
O3—S1—N2—C14	−174.2 (2)	C2—C1—C6—C7	175.1 (3)
C11—S1—N2—C14	72.0 (3)	C1—C2—C3—C4	0.8 (5)
O2—S1—C11—C10	10.4 (2)	C2—C3—C4—C5	−1.1 (6)
O2—S1—C11—C12	−170.1 (2)	C3—C4—C5—C6	0.4 (5)
O3—S1—C11—C10	142.8 (2)	C4—C5—C6—C1	0.5 (5)
O3—S1—C11—C12	−37.7 (2)	C4—C5—C6—C7	−175.4 (3)
N2—S1—C11—C10	−105.9 (2)	C1—C6—C7—N1	2.4 (4)
N2—S1—C11—C12	73.6 (2)	C5—C6—C7—N1	178.2 (3)
C16—O4—N3—C14	−0.4 (3)	N1—C8—C9—C10	−177.1 (2)
N3—O4—C16—C15	−0.1 (3)	C13—C8—C9—C10	−2.7 (4)
N3—O4—C16—C17	−180.0 (3)	N1—C8—C13—C12	176.3 (2)
C8—N1—C7—C6	−170.2 (3)	C9—C8—C13—C12	2.1 (4)
C7—N1—C8—C9	−147.2 (3)	C8—C9—C10—C11	1.5 (4)
C7—N1—C8—C13	38.6 (4)	C9—C10—C11—S1	179.81 (19)
S1—N2—C14—N3	−152.9 (2)	C9—C10—C11—C12	0.3 (4)
S1—N2—C14—C15	29.5 (4)	S1—C11—C12—C13	179.6 (2)
O4—N3—C14—N2	−177.3 (2)	C10—C11—C12—C13	−0.9 (4)
O4—N3—C14—C15	0.7 (3)	C11—C12—C13—C8	−0.3 (4)
O1—C1—C2—C3	179.8 (3)	N2—C14—C15—C16	177.0 (3)
C6—C1—C2—C3	0.2 (5)	N3—C14—C15—C16	−0.7 (3)
O1—C1—C6—C5	179.6 (3)	C14—C15—C16—O4	0.4 (3)
O1—C1—C6—C7	−4.6 (4)	C14—C15—C16—C17	−179.7 (3)

Symmetry codes: (i) −*x*+2, −*y*+1, −*z*+1; (ii) −*x*+1, −*y*+1, −*z*+1; (iii) *x*−1, *y*, *z*; (iv) *x*, −*y*+1/2, *z*+1/2; (v) *x*−1, −*y*+1/2, *z*+1/2; (vi) −*x*+2, *y*+1/2, −*z*+3/2; (vii) −*x*+2, −*y*+1, −*z*+2; (viii) *x*+1, *y*, *z*; (ix) *x*, −*y*+1/2, *z*−1/2; (x) *x*+1, −*y*+1/2, *z*−1/2; (xi) −*x*+2, *y*−1/2, −*z*+3/2.

### Hydrogen-bond geometry (Å, °)

**Table:**

*D*—H···*A*	*D*—H	H···*A*	*D*···*A*	*D*—H···*A*
O1—H1···N1	0.82	1.88	2.606 (3)	147
N2—H2A···N3^vii^	0.86	2.24	2.898 (4)	134
C10—H10···O1^ii^	0.93	2.53	3.313 (3)	141
C15—H15···O2	0.93	2.53	3.010 (3)	112

Symmetry codes: (vii) −*x*+2, −*y*+1, −*z*+2; (ii) −*x*+1, −*y*+1, −*z*+1.
